# Ovarian Neuroendocrine Neoplasms: Challenges and Future Perspectives

**DOI:** 10.3390/jcm14248833

**Published:** 2025-12-13

**Authors:** Valentina Di Vito, Gabriele Veroi, Laura Rizza, Francesca Rota, Andrea Baiocchini, Maria Cristina Macciomei, Carla Lubrano, Anna La Salvia, Andrea Lania, Lucia Rosalba Grillo, Silvia Migliaccio, Guido Rindi, Roberto Baldelli

**Affiliations:** 1 Department of Endocrinology, San Camillo-Forlanini Hospital, 00152 Rome, Italy; valentinadivito2@gmail.com (V.D.V.); gabriele.veroi@uniroma1.it (G.V.); laura_rizza@hotmail.it (L.R.); francescarota73@tiscali.it (F.R.); 2 Department of Experimental Medicine, Section of Medical Pathophysiology, Food Science and Endocrinology, Sapienza University of Rome, 00185 Rome, Italy; carla.lubrano@uniroma1.it (C.L.); silvia.migliaccio@uniroma1.it (S.M.); 3 Department of Anatomic Pathology, San Camillo-Forlanini Hospital, 00152 Rome, Italy; abaiocchini@scamilloforlanini.rm.it (A.B.); mmacciomei@scamilloforlanini.rm.it (M.C.M.); lrgrillo@scamilloforlanini.rm.it (L.R.G.); 4 National Center for Drug Research and Evaluation, National Institute of Health (ISS), 00161 Rome, Italy; anna.lasalvia@iss.it; 5 Department of Endocrinology, Diabetology and Andrology, IRCCS Humanitas Research Hospital, 20089 Rozzano, Italy; andrea.lania@hunimed.eu; 6 Department of Anatomic Pathology, Università Cattolica del Sacro Cuore, 00168 Rome, Italy; guido.rindi@policlinicogemelli.it

**Keywords:** ovarian neuroendocrine neoplasms, carcinoid tumors, neuroendocrine carcinomas

## Abstract

**Background**: Ovarian neuroendocrine neoplasms (O-NENs) are extremely rare, representing less than 1% of all ovarian neoplasms and under 5% of all neuroendocrine tumors (NETs). They encompass two primary histological subtypes: well-differentiated carcinoids and poorly differentiated neuroendocrine carcinomas, which display distinct biological behaviors and prognoses. The ovary can also be a site of metastasis from extra-ovarian NETs. Owing to their rarity, clinical management lacks standardization, and diagnosis is often incidental following surgery for presumed epithelial ovarian neoplasms. **Objectives**: This review aims to provide an updated synthesis of current evidence on the epidemiology, pathogenesis, clinical presentation, diagnosis, treatment strategies, and prognosis of O-NENs, highlighting unmet clinical needs. **Methods**: A literature search was performed on PubMed for the years 2014–2024 using the keywords: “ovarian neuroendocrine tumor”, “ovarian neuroendocrine neoplasm”, “ovarian neuroendocrine carcinoma”, and “ovarian carcinoid”. Only articles published in English were considered. Given the rarity of the disease, in addition to meta-analyses and systematic reviews, relevant case reports and case series were also included to provide a comprehensive clinical picture, yielding 32 eligible articles. **Results**: Evidence indicates that O-NENs remain understudied, with most data derived from case reports and small series. Clinical presentations vary from asymptomatic masses to hormone-related syndromes, often mimicking other ovarian pathologies. Diagnostic work-up typically follows the same protocol as epithelial ovarian cancer, with the neuroendocrine nature only recognized postoperatively. Treatment strategies are empirical and largely extrapolated from extra-ovarian NETs due to the absence of specific guidelines. Prognosis varies widely depending on histotype, stage, and secretory activity. **Conclusions**: O-NENs pose significant diagnostic and therapeutic challenges due to their rarity and heterogeneity. Greater clinical awareness, multidisciplinary management, and multicenter research are essential to establish evidence-based protocols and improve patient outcomes.

## 1. Introduction

Neuroendocrine neoplasms of the ovary (O-NENs) are rare, accounting for less than 1% of all ovarian neoplasms and less than 5% of all NETs. We distinguish two main histotypes of O-NENs: carcinoid, well-differentiated, and carcinoma, poorly differentiated, which have substantial differences in biological behavior and thus prognosis (good and poor prognosis, respectively). In addition, the ovaries may be the site of metastasis of neuroendocrine neoplasms from other organs.

However, they all have in common the poor definition of their clinical management caused by their rarity. In most cases, the neuroendocrine nature of these neoplasms is revealed only during histologic examination, while the diagnostic procedure preceding surgery is the same as an epithelial ovarian neoplasm; therapy is totally empirical. Although interest in this neoplasm has been growing in recent years, mostly case reports or case series with a small number of patients are reported in the literature; given the lack of data on large populations, it is not possible to define a shared management strategy for this pathology yet.

The impact of O-NENs on patients’ quality of life is significant and may include symptoms related to the disease (e.g., abdominal distension and pain, diarrhea or constipation, hormonal disturbances) and side effects of treatments; prognosis can be good or very poor, even in the early stages of the disease.

Thereby, a thorough understanding of O-NENs is crucial for improving early diagnosis, therapeutic management, and prognosis for affected patients. This review aims to provide a comprehensive and up-to-date overview of the epidemiology, pathogenesis, clinical manifestations, diagnosis, treatment, and prognosis of the disease.

Due to their rarity and the variability in clinical presentation, ovarian neuroendocrine neoplasms (O-NENs) represent a considerable challenge in both diagnosis and treatment. In the absence of standardized management guidelines, clinical decisions are often tailored to individual cases. Enhancing clinical awareness, promoting multidisciplinary collaboration, and advancing research—especially through multicenter studies involving larger patient populations—are crucial steps toward establishing evidence-based protocols and improving patient outcomes.

## 2. Materials and Methods

### 2.1. Search Strategy and Information Sources

We conducted a structured literature search of the PubMed database for articles published between 1 January 2014 and 31 December 2024. Search terms were chosen to capture the entire spectrum of ovarian neuroendocrine disease, with the final search terms being “(ovarian OR ovary) AND (neuroendocrine) AND (tumor OR neoplasm OR carcinoma OR cancer)” and “(ovarian OR ovary) AND (carcinoid)”. No differences in the number of retrieved articles were observed when alternating the lexical variants of the search terms, including “tumor/tumour/tumors/tumours”, “neoplasm/neoplasms”, and “carcinoma/carcinomas”. Additional records were identified through manual screening of reference lists of retrieved articles and key review papers. The search was restricted to articles published in English and studies involving human subjects.

### 2.2. Eligibility Criteria

We included meta-analyses, systematic reviews, narrative reviews, case series, and original studies that provided clinically or pathologically relevant data on ovarian neuroendocrine neoplasms (epidemiology, histopathology, clinical features, diagnosis, management, and prognosis). Exclusion criteria were conference abstracts without full text, non-English articles, animal or in vitro studies, and publications lacking primary data or clear methodological description. Duplicate publications and articles not focused on ovarian neuroendocrine disease were excluded.

### 2.3. Study Selection

Titles and abstracts retrieved from the initial search were independently screened by two reviewers. Full texts were obtained for studies meeting inclusion criteria or when eligibility was uncertain from the abstract. Discrepancies between reviewers were resolved by discussion and, when required, by consultation with a third reviewer (R.B.). The final selection included 32 articles (meta-analyses, systematic and narrative reviews, and selected original studies) that formed the basis of this review.

### 2.4. Data Extraction and Items

From each included article we extracted: bibliographic details (authors, year, journal), study design, population characteristics (sample size, age), tumor subtype(s), diagnostic methods, immunohistochemical and molecular findings, treatment modalities, follow-up duration, and main outcomes (survival, recurrence, functional syndromes). Data extraction was performed independently by two reviewers using a standardized data collection form; extracted data were cross-checked for accuracy.

### 2.5. Data Synthesis

Given the heterogeneity of study designs, reporting formats, and small patient numbers in many primary reports, a quantitative meta-analysis was not feasible. We therefore performed a structured narrative synthesis stratified by histological subtype (well-differentiated carcinoid vs. poorly differentiated neuroendocrine carcinoma), primary versus metastatic ovarian involvement, diagnostic approach, and therapeutic strategy.

### 2.6. Reporting

**Figure 1:**
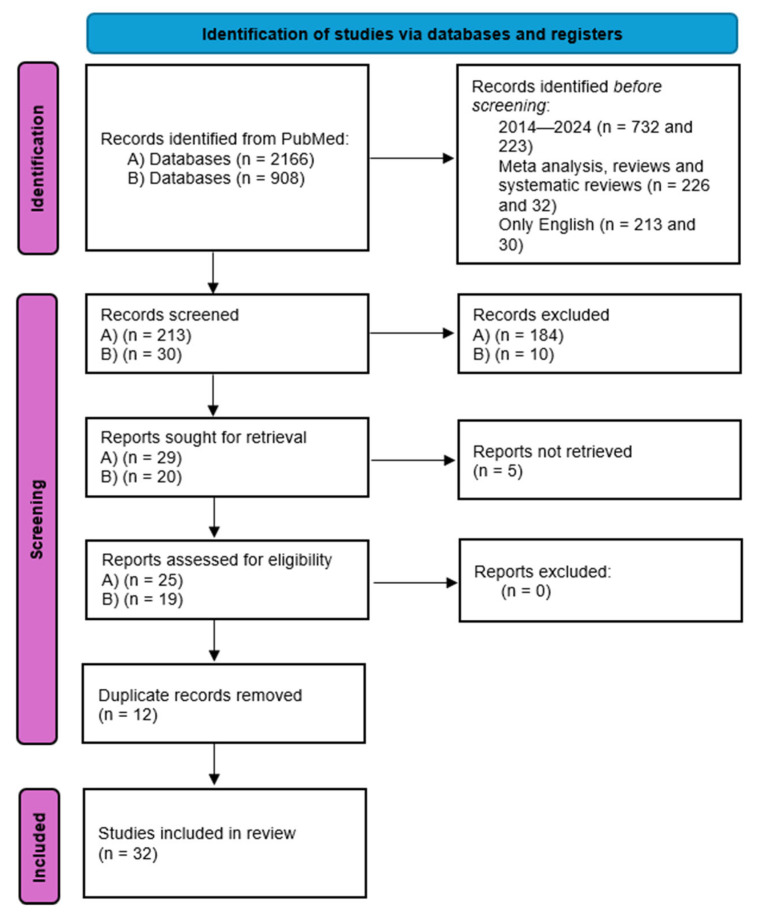
PRISMA flow diagram: 1. Identification: Records identified through PubMed searching with “(ovarian OR ovary) AND (neuroendocrine) AND (tumor OR neoplasm OR carcinoma OR cancer)” (n = 2165); Records identified through PubMed searching with “(ovarian OR ovary) AND (carcinoid)” (n = 908); Additional records identified through other sources (reference lists, manual search) (n = 1). 2. Screening: (A) Records after filters were applied (2014–2024, meta-analysis, reviews and systematic reviews, only English) n = 213; (B) Records after filters were applied (2014–2024, meta-analysis, reviews and systematic reviews, only English) n = 30; Records selected at title/abstract screening (n = 28); Records selected at title/abstract screening (n = 20); Records after duplicates removed (n = 36). 3. Eligibility: Full-text articles assessed for eligibility (n = 32); Full-text articles excluded, (n = 0). 4. Included: Studies included in qualitative synthesis (n = 32).

The review follows PRISMA 2020 reporting guidance for literature reviews. A PRISMA flow diagram summarizing the search and selection process is provided (Figure 1).

## 3. Results

### 3.1. Ovarian Neuroendocrine Neoplasms (O-NENs)

#### 3.1.1. Epidemiology

A Neuroendocrine Neoplasm (NEN) is a type of tumor that originates from neuroendocrine cells, derived from the neuroectoderm, neural crest, and endoderm; they have characteristics of both nerve and endocrine cells, and can produce and secrete neuropeptides, amines, and hormones. These tumors can arise in various organs, mainly from the gastrointestinal tract, lungs, and pancreas. NENs originating from the female genital tract account for about 2% of all gynecologic malignancies [1]. Neuroendocrine neoplasms of the ovary (O-NENs) are quite rare: according to most studies, they constitute less than 1% of all ovarian malignancies [2,3] and less than 5% of all neuroendocrine tumors [4,5,6]; some authors describe this type of neoplasm as even more uncommon, accounting for less than 0.1% of all ovarian neoplasms and for less than 1% of all carcinoid tumors [7,8]. In a 2020 study by Crane et al. the population with neuroendocrine neoplasia in the ovary accounted for 16 percent of all primary gynecologic NENs, preceded by 54 percent in the cervix and 24 percent in the uterine body [2].

#### 3.1.2. Cellular Origin

Neuroendocrine cells in the ovary may arise from the following [4,9,10]:

- Mature teratomas: NENs arising from mature teratomas (dermoid cysts) represent the most common subtype (generally, carcinoids).
- Primary differentiation: tumors originating directly from ovarian tissue without an associated teratoma; they are primary ovarian carcinoids (POC) or carcinomas; they are generally unilateral.
- Secondary metastases: frequently bilateral and associated with carcinomatosis, these are tumors metastasizing from extra-ovarian NENs, mainly lung and gastro-entero-pancreatic (GEP) NENs.

Immunohistochemical examination (IHC) is critical for the diagnosis and classification of NENs, particularly to differentiate primary ovarian NENs from neuroendocrine ovarian metastases [11,12].

#### 3.1.3. Histogenesis

To date, the histogenesis of O-NENs is unknown, but many authors have suggested different hypotheses: according to the main one, the neuroendocrine cells normally present in the epithelium of the female genital tract may be the origin of neuroendocrine tumors of the ovary. Another hypothesis proposes that primitive endocrine cells possess the ability to differentiate into multiple cell types, encompassing endocrine, non-endocrine, and neuroendocrine lineages. The last hypothesis proposes that non-neuroendocrine cells may activate genes promoting neuroendocrine differentiation and consequently develop an ovarian neuroendocrine tumor [13,14].

#### 3.1.4. WHO Classification

The 2022 WHO classification of neuroendocrine neoplasms is the latest: it recommends abandoning the term “carcinoid” and differentiating NENs in neuroendocrine tumors (NETs, well-differentiated) and neuroendocrine carcinomas (NECs, poorly differentiated) [15]. However, since there is no specific section for NENs of the ovary, the 2020 WHO terminology is still widely used.

In the 2020 WHO classification of neuroendocrine neoplasms, ovarian NENs (O-NENs) were not considered as a distinct group separate from other ovarian tumors, in contrast to NENs arising in other organs [3,11]. Instead, the WHO classification of Female Genital Tract Tumors organizes ovarian neoplasms primarily by their presumed cell of origin (e.g., epithelial, germ cell), and O-NENs are not assigned a dedicated category analogous to the classifications used for neuroendocrine tumors of the lung or gastrointestinal tract. Rather, they are typically mentioned within the context of other tumor entities—such as teratomas—or described as rare variants. This absence of a specific, standardized category for O-NENs within the primary gynecological taxonomy is one of the key gaps our manuscript aims to underscore. So, according to this classification, based on Ki-67% index, mitotic count, and necrosis, the two histotypes of ovarian NENs are “carcinoid” (Grade 1) and “carcinoma” (Grade 3) [2].

A significant diagnostic challenge arises from the application of grading systems designed for other sites. In the 2022 WHO classification of neuroendocrine neoplasms, a well-differentiated NET with a Ki-67 index above 20% would be classified as a NET, G3. However, in the context of the ovary, where the distinction is often simplistically made between low-grade ‘carcinoid’ and high-grade ‘carcinoma’, such a tumor could be misclassified as a neuroendocrine carcinoma (NEC). This lack of a specific, nuanced classification for O-NENs can lead to diagnostic inconsistencies and complicates the comparison of clinical data. Future classifications should aim to integrate the ovarian-specific context with the universal NEN nomenclature.

The previous WHO tumor classification, now abandoned, dates back to 2014: it described carcinoids as “monodermal teratoma and somatic-type tumors from a dermoid cyst”, remarking on its histological origin, and small cell ovarian NEC pulmonary type as “miscellaneous tumors”.

The main differences between the classifications are shown in Table 1 [2,3,6].

### 3.2. Ovarian Carcinoid

#### 3.2.1. Epidemiology

Ovarian carcinoid is the well-differentiated form of O-NENs; it was described for the first time in 1939 by M. J. Stewart in a case report of a 68-year-old woman who reported the presence of blood and mucus in her stool in absence of pain and/or tenesmus [1,11].

Ovarian carcinoid constitutes less than 1% of all carcinoids [16]; it can affect women of all ages, ranging from 14 to 83 years old [11], with a mean age at 53 years old; however, most studies in the literature agree that ovarian carcinoid most frequently affects perimenopausal and postmenopausal women [1,11,12,16,17].

#### 3.2.2. General Features

“Ovarian carcinoids are often found in association with teratomas, which are common gynecological lesions.” As reported by Soga et al., this coexistence is observed in about 57% of cases [11], while according to Bidzinski et al. up to 75% of ovarian carcinoid cases are associated with a teratoma [10]; they include mature cystic teratomas, immature (malignant) teratomas, and monodermal (specialized) teratomas [2,16].

Albeit rarely, small carcinoids can be identified in other ovarian neoplasms such as germ cell neoplasms, yolk sac tumors, Brenner or Sertoli–Leydig tumors [6,12].

Even rarer is the association of two tumor formations within the same teratoma: Ayyanar P. et al. describe the case of a patient with a single teratomatous formation of the left ovary, which, once removed, was subjected to histological examination; it was found to be a synchronous colonic adenocarcinoma and a carcinoid, both matured inside the same mature cystic teratoma [18].

On the other hand, in the absence of teratomas, ovarian carcinoids may be differentiated in the following [2,10]:

- Primary (POC), thus originating directly from ovarian tissue;
- Secondary, i.e., metastasis of neuroendocrine neoplasm with extra-ovarian primitivity, most frequently originating from the midgut or respiratory tract.

The primary form accounts for 0.1% of all malignant ovarian neoplasms and is more frequent than secondary forms [2,12,16]; early differential diagnosis between the primary and metastatic forms is of paramount importance as the latter is associated with worse symptoms, lower survival rates, and high 5-year mortality rates [16].

The scientific literature indicates that primary ovarian carcinoid (POC) typically presents as a small, solid, lobulated, unilateral mass. In contrast, the metastatic form is more often multinodular, with bilateral ovarian involvement, extra-ovarian spread, and an absence of teratomatous elements [1,2,6,16].

**Figure 2:**
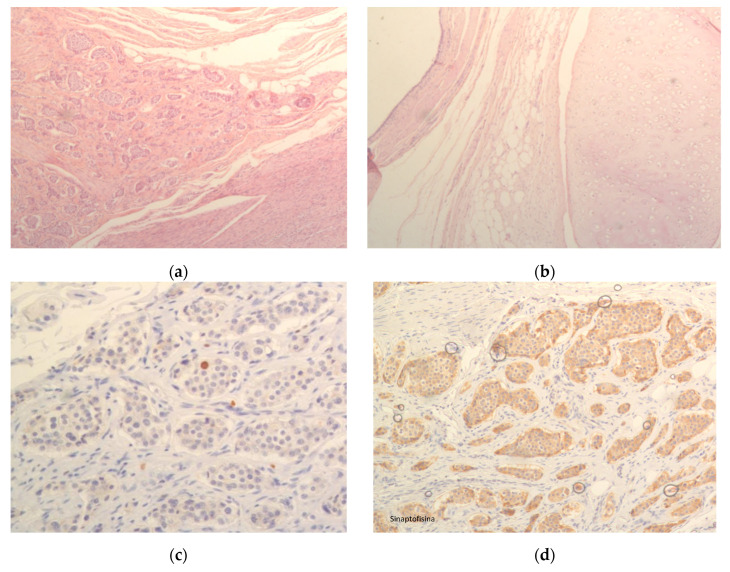
(**a**) Microscopic section of ovary; (**b**) Microscopic section of teratoma; (**c**) Positivity for Ki67; (**d**) Positivity for synaptophysin.

In Figure 2, we present a representative case, showing a microscopic section of the ovary, a microscopic section of the teratoma, as well as positive staining for Ki-67 and synaptophysin (Figure 2).

#### 3.2.3. Histological Classification and Microscopical Description

There are four histotypes of primary ovarian carcinoid in the literature, with very similar characteristics to their gastrointestinal tract counterparts [2,10,11,12]:

1. Insular: the cells are uniform and polygonal, with an abundant basophilic or amphophilic cytoplasm, eosinophilic granules, and a centrally located, round or oval nuclei with salt and pepper chromatin; they are arranged in small acini or tubular glands, organized as small groups in fibrous stroma in which solid nests can be seen;
2. Trabecular: the neoplastic cells are regular, with nuclear features similar to the insular histotypes; the are displayed in parallel ribbons, cords, or trabeculae, surrounded by fibrous stroma [5];
3. Strumal: it is composed of insular or trabecular histotype and thyroid tissue; the different components can be clearly separated or mixed (the thyroid follicles can be found among carcinoid cells or the proliferated neuroendocrine neoplastic cells can cover some follicles) [19];
4. Mucinous (Goblet cells, carcinoid): the cells are uniform, cuboidal to columnar, filled with mucin vacuoles and small and round to compressed nuclei (similar to an appendix NEN microscopically); it is characterized by the presence of numerous small glands or acini with very small lumen; rarely, an atypical or even a carcinomatous component can be associated with the mucinous histotypes, the latter being characterized by a large island of tumor cells or closely packed glands with severe histologic atypia, and increased mitosis and necrosis [20].

The insular histotype is the most frequent, especially in Western countries; it derives from the middle intestine and manifests with carcinoid syndrome in approximately one-third of cases [21]. The classic carcinoid syndrome, characterized by flushing, diarrhea, and bronchospasm, is the most commonly reported paraneoplastic syndrome. According to Mishra P. et al., in patients with carcinoid syndrome of the insular histotype, releasing various biogenic amines and polypeptides directly into systemic circulation may cause carcinoid heart syndrome, a right-sided valvular dysfunction and right ventricular failure, in half of the patients [9]. In contrast, the trabecular and strumal histotypes are less frequently associated with the classic syndrome but may cause severe constipation due to the production of peptide YY. Overall, the majority of ovarian carcinoids are non-functioning, with symptoms more often related to the mass effect of the tumor.

The trabecular and strumal histotypes are primarily observed in Japan and arise from the upper or lower intestine; clinically, these two generally cause constipation by producing polypeptide YY, which inhibits intestinal motility; once the carcinoid was removed, the symptoms generally regressed without further medical therapy [21,22,23].

The stromal histotype can, sometimes, produce different hormones, like androgens (causing virilism, hirsutism, androgenic type of alopecia, increased pilosity, and clitoromegaly), estrogens (causing endometrial hyperplasia and abnormal bleeding), or even thyroid hormones (causing hyperthyroidism) [19].

The insular, trabecular, and strumal histotypes generally have a more favorable prognosis. In contrast, the mucinous histotype, although rare, is more frequently associated with pelvic dissemination and metastatic disease, despite the limited number of cases described. Hsu et al. reported the case of a 33-year-old patient who underwent fertility-sparing staging surgery, including left salpingo-oophorectomy, which revealed a mucinous carcinoid with focal carcinomatous changes, and a right ovarian biopsy, which showed no malignancy. Six years later, she was hospitalized for removal of a 15 cm right ovarian mass, which was found to be a carcinomatous mucinous carcinoid with metastases to multiple abdominal organs. She received nine courses of chemotherapy but died 86 months after the new diagnosis. Another case involved a 39-year-old woman who underwent unilateral adnexectomy for a mucinous ovarian carcinoid, only to develop metastasis in the contralateral ovary ten years later [20].

#### 3.2.4. Diagnosis

The first step for the diagnosis of carcinoid is the imaging: generally, patients discover the presence of ovarian masses either incidentally, during routine checkups, or because they complain of various symptoms, like abdominal or pelvic pain, bloating, discomfort, or abnormal vaginal bleeding and, depending on which substance is produced, changes in bowel habits (diarrhea in the case of carcinoid syndrome, constipation when PYY is produced) [7,24]. Sometimes, as mentioned before, these tumors can produce different hormones: a case report from 2014 describes a patient with Cushing’s syndrome, which turned out to be due to ectopic ACTH secretion from a carcinoid tumor within an ovarian mature teratoma; 1 year after the teratoma and the carcinoid tumor were removed, the syndrome had disappeared and the patient was permanently cured [25].

Moreover, it is interesting to point out that, albeit rarely, the patient with ovarian carcinoid could be found to have carcinoid syndrome even in the absence of liver metastasis due to direct drainage of the ovarian vein into the inferior cava [1].

Pelvic or transvaginal ultrasound exams generally identify the presence of teratomatous elements, and describe these masses as anechoic ovarian solid cysts, sometimes multilocular [8]. A CT scan is generally required after an initial identification of these masses, and secondary exams like MRI and PET-CT scan with [68Ga]-DOTA-TATE can help to a complete disease staging [17].

Imaging features are non-specific. On ultrasound, ovarian carcinoids often appear as solid, well-defined, lobulated masses, which may contain cystic areas. They can be identified within a teratoma, which itself has characteristic imaging findings. CT or MRI may be used for further characterization and staging [11].

The diagnostic use of serum tumor markers is reported in the minority of studies, and the data is conflicting: the classic ovarian markers like CA19.9, CEA, and CA-125 can vary greatly, as well as the neuroendocrine markers, such as chromogranin A and NSE, since they are either reported as elevated, or within the normal range [3,8].

On the other hand, IHC is critical for the diagnosis, in particular to differentiate primary disease from secondary metastases: chromogranin-A (diagnostic of a neuroendocrine tumor), synaptophysin, and CD56 are the most commonly used neuroendocrine markers; secondary markers are a wide spectrum cytokeratins (CKs).

Other IHC markers are fundamental in order to distinguish between primary and metastatic tumors: thyroid transcription factor-1 (TTF-1) for the thyroid and lung (but it can be positive in strumal carcinoids), ISL-1 and PDX-1 for the pancreas, PAX8 for the thyroid, and CDX-2 and villin for the gastrointestinal tract [11,12].

#### 3.2.5. Prognosis

The prognosis of ovarian carcinoid is generally good as it remains confined to the ovary and is diagnosed at an early stage; in most cases, patients are candidates for surgical resection, and chemotherapy and radiotherapy are not necessary [1].

The survival rate is generally good: the 10-year survival rate in stage I patients is close to 100% according to a review from 2020 [7]; according to Virakar et al., the 5-year survival rate of patients with ovarian carcinoid is a little lower, 84% or 94% depending on whether or not it is associated with teratoma [2]. The prognostic factor seems to be Ki67 in these tumors: in a retrospective study, the authors compared nine patients with primary ovarian carcinoid with twenty-seven patients with metastatic ovarian carcinoid reporting significantly higher median Ki67 values in the latter (9.7% vs. 2.3%) and significantly lower median survival rates (5.8 years vs. 14.2 years) [16].

#### 3.2.6. Therapy

The therapy of choice in ovarian carcinoids is surgical resection with curative intent; in fact, most carcinoids are diagnosed at an early stage by being confined to the ovary [1,11,26].

In young patients with a desire for motherhood and with early stage ovarian disease (stage I), the authors agree in preferring fertility-sparing surgery, such as unilateral salpingo-oophorectomy, as this stage is generally characterized by a unilateral lesion and good prognosis [1,7,11,21,27].

Instead, premenopausal and postmenopausal women generally undergo bilateral salpingo-oophorectomy and hysterectomy [1].

In a 2023 review, the authors argued that, given the absence of controlled trials in this regard, unilateral adnexectomy without hysterectomy cannot be considered a validated surgical strategy and therefore the treatment of choice should remain bilateral salpingo-oophorectomy and hysterectomy, with surgical debulking in case of extra-ovarian disease spread and/or metastasis [11].

It is in any case essential to perform accurate pre- and postsurgical staging in order to recognize any occult metastases [1,11].

In the presence of liver metastases, these should also be surgically removed or, if unresectable, treated with target therapies such as cryotherapy and radiofrequency [27]; the use of hepatic artery embolization/chemoembolization with streptozotocin in the treatment of carcinoid liver metastases has shown response rates of up to 67% with median overall survival (OS) of 31 months [1]. In advanced stages of disease (II-III-IV), complete staging is essential, and the treatment of choice should be bilateral hysterectomy with bilateral salpingo-oophorectomy [7,27].

This type of surgery should also be used in the presence of insular, trabecular, and mucinous ovarian carcinoid; in the mucinous subtype, it may also be appropriate to perform an omentectomy with para-aortic lymphadenectomy given the tendency of this tumor to spread via the lymphatic pathway [1,7].

Somatostatin analogs such as Octreotide and Lanreotide may be useful in the presence of functioning carcinoids in patients with carcinoid syndrome.

There is consensus in the literature that there is no scientific validation of the use of adjuvant therapies in ovarian carcinoid patients even though, in clinical practice, radiotherapy and adjuvant chemotherapy treatments are often used in stage III and IV ovarian carcinoids [1,11,27]. Data on the efficacy of adjuvant therapy are derived from heterogeneous, retrospective studies and should be interpreted with caution. One review compared patients receiving surgery plus adjuvant chemotherapy to those treated with surgery alone [26]. However, the groups were not matched for stage or histology; notably, 60% of the chemotherapy group had distant metastases at diagnosis. Consequently, while recurrence rates were lower in the chemotherapy group, overall survival was not higher, likely reflecting the more advanced disease in this cohort. There is no robust evidence to support the routine use of adjuvant chemotherapy in early-stage disease. In another paper published in 2020, two patients with primary ovarian carcinoid, one of whom received adjuvant chemotherapy for the development of metastases in the postoperative period, were compared: the authors reported a clinically well condition and good prognosis during follow-up in both patients [7].

In contrast, there are currently no reliable data on the role of molecularly targeted therapies, VEGF, and mTOR inhibitors in the treatment pathway of patients with female genital tract carcinoid [1].

Finally, for patients with advanced, well-differentiated, somatostatin receptor-positive metastatic disease, Peptide Receptor Radionuclide Therapy (PRRT) with [^177^Lu]Lu-DOTA-TATE may be a viable treatment option, extrapolated from its established success in gastro-entero-pancreatic NETs.

### 3.3. Ovarian Neuroendocrine Carcinoma

#### 3.3.1. General Epidemiology

Ovarian neuroendocrine carcinomas (O-NECs) are the poorly differentiated and aggressive form of O-NENs. They are generally characterized by a high mitotic index and necrosis, focal or extensive, with extra-ovarian pelvic spread and ascites at diagnosis in almost all cases [13,28].

The hypothesis regarding the histogenesis of O-NECs is the same for ovarian NETs in general: either a neoplastic transformation of mature neuroendocrine cells into ovarian neuroendocrine tumors; or a neoplastic neuroendocrine transformation of non-neuroendocrine cells, due to gene sequence activation, similar to that of neuroendocrine cells; or some neuroendocrine tumors are believed to originate directly from teratomatous components. The last hypothesis suggests that pure small cell carcinoma of the ovary (SCCO), a histological form of O-NEC, may directly develop from normal ovarian tissue [14].

There are two distinct histological forms of ovarian neuroendocrine carcinoma [10,12]:

1. “Small cell” carcinoma of the ovary (SCCO);
2. “Non-small cell” or “large cell” neuroendocrine carcinoma of the ovary (LCNEC or LCNEO).

#### 3.3.2. Small Cell Carcinoma of the Ovary (SCCO)

The small cell form was first identified in 1979 by Reed et al. and it constitutes less than 1% of all ovarian malignancies [3,29].

We distinguish two subtypes of SCCO:

- Small cell carcinoma of the ovary hypercalcemic type (SCCOHT)
- Small cell neuroendocrine carcinoma of the ovary, pulmonary type (SCNEC-PT).

SCCOHT is no longer considered to belong to the family of neuroendocrine neoplasms because it appears to be associated with a germline or somatic mutation in the gene encoding for SMARCA4, an inactivating mutation responsible for reduced BRG1 protein expression and frequently found in rhabdoid tumors.

For this reason, the scientific literature now agrees that SCCOHT is a malignant rhabdoid tumor rather than a neuroendocrine carcinoma of the ovary [2,3,30].

Its discussion, therefore, is beyond the scope of the review topic.

#### 3.3.3. Epidemiology of SCOOPT

The identification of the lung subtype dates back to 1992 when Einchorn et al. introduced the concept of SCCOPT: it is a rare tumor, extremely aggressive and with a poor prognosis even at early stages. Its incidence is less than 1% of all ovarian malignancies, affecting postmenopausal women according to most studies [12] with a mean age of 51 years [2,3]. This finding is confirmed in a retrospective review of 63 cases of SCCOPT published in 2025 in which the authors report a mean age of 52.4 years, with a wide range of 14–85 [3]; yet, another paper from 2021 describes an involvement by SCCOPT of women also in the perimenopausal age [27].

#### 3.3.4. Microscopical Description of SCOOPT

Microscopically, it is similar to pulmonary small cell carcinoma: the tumor cells are small, have round, ovoid, or slightly spindled hyperchromatic nuclei, often with a “salt-and-pepper” chromatin and molding and scant cytoplasm. They are organized in sheets and nests, with an abundant mitotic activity, frequent apoptosis, and extensive necrosis [2,3,14,27].

As with carcinoids, SCCOPT often appears within mature cystic teratomas or other tumor formations [14]. The frequent finding of association with other tumors has led to the hypothesis that SCCOPT may originate from the dedifferentiation of other tumors of a non-neuroendocrine nature; in a 2021 study, the authors reported that only 40% of the described cases of SCCOPT were a pure form while the remaining 60% were associated with epithelial tumors such as endometrioid carcinomas, mucinous tumors, and Brenner’s tumors [27]. Another study reports this association in 30.16% of patients surveyed [3]. Ikota and coauthors described the case of a patient with mature ovarian cystic teratoma who underwent malignant transformation whose histology described the association of teratoma with four tumors: SCCOPT, adenocarcinoma, squamous cell carcinoma, and transitional cell carcinoma [3]. This association of neuroendocrine and non-neuroendocrine tumors is defined as Mixed neuroendocrine/non-neuroendocrine neoplasms (MiNENs) [27].

#### 3.3.5. Signs and Symptoms of SCOOPT

According to most authors, SCCOPT presents bilateral ovarian involvement at diagnosis in half of the cases [2,27]; in contrast, a recent review in 2024 reported bilateral ovarian involvement in 24% of 63 reviewed patients with SCCOPT, with metastatic disease at diagnosis in 73% of them [3]. The organs most frequently involved by metastasis were found to be lymph nodes, omentum, peritoneum, colon, brain, liver, and uterus [3].

The onset symptoms reported by patients with SCCOPT are abdominal pain, constipation, vaginal bleeding, nausea, anorexia, and dysuria but also the presence of ascites and/or abdomino-pelvic masses [3,24]. The presence of paraneoplastic syndrome is not clear: according to He et al., it is a rare occurrence, with only one case of SCCOPT with inappropriate ADH secretion syndrome described in the literature [3]; otherwise, a review of 63 cases with O-NEN from 2024 identified paraneoplastic syndrome in nine patients out of fifty with neuroendocrine carcinoma (18%); in particular, due to findings like hypercalcemia, loss of color vision, uveitis, and bilateral frontal lobe ischemia were identified due to the production of auto-antibodies by the tumor [24].

#### 3.3.6. Diagnosis of SCOOPT

The diagnostic procedure involves instrumental investigations in the first instance. SCCOPT presents at the ultrasound as a solid-cystic mass with irregular margins, richly vascularized in the context of ascites. Key examinations for disease staging are abdominal MRI with contrast medium, which allows for loco-regional staging, and CT and PET-CT, either with FDG or 68Ga-DOTA-TATE, which are useful in identifying distant metastases; at present, no specific mutations or molecular markers of SCCOPT have been identified [3].

The definitive diagnosis of SCCOPT is postsurgical and is based on the histologic and IHC features of the excised lesion or sometimes the section of tissue obtained by ultrasound-guided biopsy [3].

Immunohistochemistry of SCCOPT generally demonstrates positivity for at least one of the neuroendocrine markers among chromogranin A, CD56, neuron-specific enolase, synaptophysin, CK AE1/AE3, and CK 20 (dot-like); generally, vimentin, TTF-1, inhibin, S-100, WT-1, estrogen receptor (ER), and progesterone receptor (PR) are negative [12]. In the different studies that evaluated the IHC profile of these neoplasms, chromogranin A was the most frequently expressed marker with positivity rates above 50% [2,3]. Ki67 was greater than 30% in all SCCOPT patients reported in a 2024 study, and greater than 70% in 44% of them [3].

Finally, from a molecular viewpoint, SCCOPT does not show a consistent specific mutation. This contrasts with SCCOHT, which is characterized by the inactivating mutation of the SMARCA4 gene, and ovarian metastatic lesions from other primaries (e.g., lung), which may show mutations in genes like TP53 and RB1. The absence of a SMARCA4 mutation is a key diagnostic tool to rule out SCCOHT.

#### 3.3.7. Differential Diagnosis of SCOOPT

The main differential diagnoses of SCCOPT are SCCOHT, LCNEC (see LCNEC chapter), and ovarian metastasis from small cell cancer, typically lung (“Small cell” lung carcinoma, SCLC).

Most studies agree that the mean age of onset of SCCOPT is higher than that of SCCOHT (50 vs. 24 years) and ovarian metastasis from SCC (50 vs. 47 years) [2,3,27].

At onset, SCCOPT’s ovarian involvement is bilateral in most cases in contrast to SCCOHT, which generally occurs in only one ovary; ovarian metastases from SCC typically present as multinodular masses in both ovaries and lymphovascular invasion [2,3,27]. In SCCOHT, hypercalcemia is present at onset in more than 65% of cases while SCCLC is frequently associated with paraneoplastic syndrome; neither clinical syndrome is present in SCCOPT [2,3,27].

Ovarian metastases from SCC present a cytopathologic picture similar to SCCOPT except for the cell morphology that may sometimes appear fusiform rather than roundish while SCCOHT differs from SCCOPT by the presence of chromatin aggregates, prominent nucleoli, and follicle-like spaces in addition to presenting in almost 50% of large cell cases [2,3,27]. Being neoplasms of neuroendocrine nature, SCCOPT and ovarian metastases from SCC both show positive immunohistochemistry for typical neuroendocrine markers such as NSE, synaptophysin, CgA, and CD56. Instead, they show different positivity to INSM1 and TTF1, with variable expression in metastatic cells depending on the primitive, and positivity to EMA, p53, and CK20 in SCCOPT; the latter is generally negative for vimentin and TTF1 (this one generally positive in metastatic SCLC). SCCOHT, having no neuroendocrine nature, is negative for NSE, EMA, and CgA and positive for Vimentin and WT-1 [2,3,27].

Finally, also from a molecular point of view, SCCOPT does not show any specific mutation unlike SCCOHT, which is characterized by the inactivating mutation of the SMARCA4 gene, and ovarian metastatic lesions, which may instead show increased an expression of EZH2, expression of CREBBP, and inactivating mutation of EP300 [3].

#### 3.3.8. Therapy of SCOOPT

The therapy of choice for SCCOPT, mostly affecting postmenopausal women, is derived from the standards established for epithelial ovarian cancer: primarily surgery with radical intent based on total hysterectomy, bilateral adnexectomy, omentectomy, and retroperitoneal, pelvic, and para-aortic lymphadenectomy; subsequently, adjuvant therapies such as chemotherapy and radiotherapy can follow [3,29].

In premenopausal women with fertility desire and diagnosed early-stage disease, on the other hand, a unilateral adnexectomy may be considered, upon confirmation of the absence of disease in the contralateral uterus and adnexa; however, given the high risk of recurrence associated with this pathology, their removal once pregnancy is terminated is recommended [2,3]. A recent 2023 study of SCCOPT patients with localized disease compared the 5-year OS of those undergoing bilateral hysteroannessiectomy with those undergoing bilateral adnexectomy without hysterectomy, but it showed no significant difference between the two groups [3].

The prognostic role of lymphadenectomy, on the other hand, remains a matter of debate; in fact, some authors suggest a benefit brought about by this on the OS rate, while others refute this by stating that pelvic and retroperitoneal lymph node excision would not be associated with reduced mortality [3].

In a recent review published in Cancers in 2022, adjuvant cisplatin- and etoposide-based chemotherapy is indicated in ovarian neuroendocrine carcinomas as part of the treatment pathway in these patients while the absence of data in favor of neoadjuvant CHT [2] is emphasized. Because of features in common with SCLC, the commonly used chemotherapeutic drugs are cisplatin and etoposide, with replacement of cisplatin by carboplatin in case of nephro- or ototoxicity. In a very recent 2025 study of patients with SCCOPT, the authors point out the absence of significant prognostic differences between the group receiving adjuvant cisplatin + etoposide chemotherapy and the group receiving second-line adjuvant chemotherapy regimen [3].

Radiation therapy may be considered in cases of localized disease to consolidate the response achieved with chemotherapy, although it does not appear to significantly alter the overall natural history of the disease. However, the available data are conflicting. For example, Reckova et al. reported a case of a patient with metastatic ovarian neuroendocrine carcinoma who initially received radiotherapy to metastatic lesions and systemic chemotherapy, followed by pelvic surgery. Despite this multimodal approach, she developed disease progression in the bone, breast, right kidney, pelvis, brain, and lung six months later and died two years after radiotherapy [3]. Conversely, other studies have reported a 5-year overall survival of 73% and a cancer-specific survival of 70% in advanced disease [2]. As noted, the evidence remains inconsistent. Given the low incidence of these tumors, data on new therapies in SCCOPT are rare; in a 2022 study, the authors, analyzing the molecular profile of a SCCOPT, showed excessive activation of a protein kinase downstream of mTOR suggesting a potential use of mTOR inhibitor drugs in these neoplasms [3].

Immune checkpoint inhibitors (ICIs) against PD-1 and PD-L1, or the first-line treatment drugs for other ovarian cancers like anti-angiogenic drugs such as vascular endothelial growth factor (VEGF) inhibitors and tyrosine kinase inhibitors (TKI) are also promising therapies for SCCOPT: in a study published in 2022, the authors described a two-year survival following surgery and no recurrence during follow-up in a patient with brain metastases from SCCOPT treated simultaneously with apatinib (TKI) and carilizumab (PD1 antibody) [3].

#### 3.3.9. Prognosis of SCOOPT

At present, the prognosis of patients with this disease is inauspicious in the majority of cases, due to the aggressive biological behavior (in particular, advanced stage, high-tumor grade, and the omission of adjuvant therapy (surgery, chemotherapy, or radiotherapy) are linked to a lower OS) and to the failure to identify the optimal therapeutic approach of neuroendocrine carcinomas of the ovary caused by the lack of prospective studies on the subject [3,12]. A study by Legarreta et al. reported a median survival in 50 patients with neuroendocrine carcinoma of 1.6 years [24].

#### 3.3.10. Large Cell Neuroendocrine Carcinoma of the Ovary (LCNEC or LCNEO)

LCNEC is the rarest of all ovarian neuroendocrine tumors and in most cases presents at diagnosis at an advanced stage [2]. It is the second form of high-grade, poorly differentiated NEC of the ovary, and it is very aggressive; because of its characteristics it is defined by the WHO as an undifferentiated form of non-small cell neuroendocrine carcinoma and often presents in association with tumor cells of germinal or epithelial origin [13,27,28,31]; this frequent association with other tumors led to the hypothesis that these epithelial or germ cells may activate gene sequences specific to neuroendocrine cells, thus undergoing neoplastic transformation, leading to the development of an LCNEC [3]. As for the other forms of O-NENs, another hypothesis has been made for its pathogenesis, such as an origin from normal neuroendocrine cells, from teratomatous cells, or from primitive cells [28].

#### 3.3.11. Epidemiology

The mean age of the onset of LCNEC is variable: the literature is in agreement in reporting an involvement of both pre- and postmenopausal women, with the age at diagnosis ranging from 22 to 76 and 18 to 80 years, and a mean age at 70 years old [13,28]; the latter finding was confirmed by a very recent review in 2024 [3].

#### 3.3.12. Microscopical Description

The cells are poorly differentiated, the dimension is intermediate to large; they are pleomorphic, round to oval; they have abundant cytoplasm, prominent nucleoli, and coarse granular chromatin within large nuclei. They are arranged in solid, trabecular, or nested patterns. Extensive mitoses and necrosis are present [2,3,28,32].

#### 3.3.13. Signs and Symptoms

The most commonly reported onset symptoms of LCNEC are abdominal distension, discomfort or pain, a palpable mass, urinary discomfort, vaginal bleeding, especially in postmenopausal women, and ascites [13].

LCNEC can cause paraneoplastic syndromes too: in two different case reports from 2020 and from 2022, the authors reported two patients with all the typical signs of Cushing Syndrome, such as hypertension, hyperglycemia, central obesity, hyperpigmented skin, moon face, elevated serum cortisol, and adrenocorticotropin (ACTH); further investigation revealed in both patients a LCNEC with ectopic production of ACTH [3,33]. In 2004, a patient was diagnosed with LCNEC following blood test findings of hypercalcemia and elevated PTH levels [3].

Currently, probably also due to the rarity of the disease, no incidental finding of LCNEC during instrumental imaging has been described.

#### 3.3.14. Diagnosis

No specific radiological features distinguishing LCNEC are described [3,13,28,31]. Most LCNECs appear as a partially solid or partially cystic mass between 9 and 30 cm in diameter, a finding confirmed in most cases reported in the literature [3,13,31].

Imaging is the first step in the identification of the disease; pelvic and transvaginal ultrasound, Ct scan, and MRI are the most used, while secondary imaging through PET-CT is used to identify metastasis: a review in 2022 describes a patient who, following the diagnosis of LCNEC, underwent PET/CT scan with both [68Ga]-DOTA-TATE and [18F]-FDG; the dual tracer thus enabled the identification of all metastases, as some of these were even less differentiated than the original tumor and therefore, having lost somatostatin receptors, showed no uptake of [68Ga]-DOTA-TATE but a positive uptake of [18F]-FDG due to the high mitotic index [33].

IHC positivity of neuroendocrine markers allows confirmation of the neuroendocrine nature of the neoplasm; these markers are typically found to be expressed in LCNEC albeit variably from patient to patient [3,13,31]. The most commonly used markers in IHC analysis are CgA, synaptophysin, CK, and CD56 while NSE and Leu-7, lacking specificity, cannot be considered indicative of neuroendocrine differentiation in the absence of positivity of the other aforementioned markers [13].

The use of serum markers for the identification of the disease and evaluation of the response to treatment is controversial: according to a review from 2014, serum CA125 levels, that are usually linked to tumor progression or recurrence of other ovarian neoplasms, in LCNC showed a different grade of expression, regardless of the stage or the spread [13]. A review from 2020 stated that chromogranin, although a sensitive and specific serum marker for low-grade neuroendocrine tumors, has limited use in high-grade NEC, as well as for NSE, because NEC serum tumor markers have low specificity and high variability [32]. On the other side, a more recent review from 2024 states that CA125 was elevated in over 80% of the patients analyzed, so other classic tumor markers like CA199, CA724, NSE, CEA, and AFP may also be elevated and used for clinical purposes [3].

#### 3.3.15. Therapy

There is no standardized treatment for LCNEC. Surgical management follows the same principles as for SCCOPT, with decisions regarding fertility preservation guided by a thorough patient history and evaluation [3].

The choice of adjuvant chemotherapy generally depends on the presence or absence of LCNEC-associated epithelial component: in the case of pure LCNEC, the cisplatin + etoposide regimen is the first choice while in the case of predominance of the epithelial component, the choice should be paclitaxel and carboplatin [3]; for example, a 2014 study reported the case of a 67-year-old woman diagnosed with LCNEC who was found on IHC analysis to be positive for pancytokeratin, focally positive for NSE, and negative for synaptophysin and chromogranin A, who was then put on an adjuvant CHT regimen of paclitaxel 175 mg/m^2^ and carboplatin every 3 weeks, and who was disease-free 5 months after surgery [13].

Further confirmation of the efficacy of this therapeutic modality is provided to us by a case reported in a 2013 study in which the patient with LCNEC in association with mucinous adenoma and undergoing first-line adjuvant CHT based on cisplatin and etoposide developed liver progression after three cycles of chemotherapy and only 4 months after surgery [31].

The combination of adjuvant cisplatin + paclitaxel CHT is reported in some studies but would appear to be associated with reduced survival, as in a 58-year-old LCNEC patient who developed lymph node-level disease recurrence 5 months after the last cycle of this adjuvant chemotherapy regimen [3].

Tri-weekly administration of Taxotere 75 mg/m^2^ is generally used in cases of lymph node-loaded recurrence after first-line adjuvant CHT [13,27].

The combination of CHT and local adjuvant RT is a must in cases of advanced disease, as it would be associated with a 70% relative cancer survival and 73% 5-year OS [2].

There is a lack of data in the literature about the use of neoadjuvant chemotherapy [2].

Targeted therapies are currently under investigation. For instance, a recent 2024 study published in *Frontiers of Oncology* reported a 4-month progression-free survival in a woman with low HER2-expressing LCNEC treated with trastuzumab deruxtecan. This antibody–drug conjugate (ADC) combines a humanized IgG1 monoclonal anti-HER2 antibody—sharing the same amino acid sequence as trastuzumab—with DXd, an exatecan derivative and topoisomerase I inhibitor [3].

#### 3.3.16. Prognosis

Given its high malignancy and grade, and the frequent presence of metastasis at diagnosis, prognosis is poor even at early stages of the disease; neoplasm size, lymph node involvement, surgery, and chemotherapy are considered independent prognostic factors for OS of LCNEC patients [3].

In a 2019 review of the literature comparing the median survival of patients with LCNEC treated with adjuvant cisplatin-based CHT with that of patients not receiving adjuvant CHT, it was higher in the first group (48.0 vs. 9.8 months); the limitation of this analysis described by the same authors was the small sample size, which did not allow for significance of the result (*p* = 0.176) [3,28]. In the same study, the authors made a further comparison between patients with LCNEC at any stage of disease with those at stage I, describing overlapping mean OS values between the two groups, 42.4 and 42.0 months, respectively [28]. In a review from 2020, the authors described the OS of 45 patients with LCNEC at different stages, 15 at stage I, 3 at stage II, 10 at stage III, 11 at stage IV, and 6 not classified; all patients received surgery and 39 of them also received chemotherapy; at the moment of publication, median OS for all stages was 8 months, while stratified median OS was 9.5 months for stage I, 22.5 months for stage II, and 8 months for stage III and IV [32].

The prognosis of patients with LCNEC is inauspicious in most cases, and the best treatment strategy has not yet been defined due to the lack of prospective studies [3].

## 4. Discussion

Ovarian neuroendocrine neoplasms (O-NENs) represent a rare and clinically diverse subset of gynecologic tumors, accounting for a small fraction of both ovarian neoplasms and neuroendocrine tumors overall. Their rarity has significantly limited the accumulation of high-level evidence, resulting in the absence of universally accepted diagnostic and therapeutic guidelines. Current clinical management is largely extrapolated from protocols established for neuroendocrine neoplasms in other anatomical sites, particularly the gastrointestinal tract and lungs.

Preoperative diagnosis remains particularly challenging due to the non-specific clinical and radiological features of ovarian neuroendocrine neoplasms (O-NENs), which often mimic epithelial ovarian malignancies. In most cases, their neuroendocrine nature is only identified postoperatively through histopathological and immunohistochemical analysis. This highlights the importance of assessing neuroendocrine markers, such as chromogranin A, synaptophysin, and Ki-67; when evaluating atypical ovarian tumors biologically, O-NENs encompass a wide spectrum of behaviors. Well-differentiated carcinoid tumors often exhibit indolent growth and favorable prognosis, while poorly differentiated neuroendocrine carcinomas (NECs) are highly aggressive, with rapid progression and poor survival outcomes. Accurate grading using the Ki-67 index, and careful distinction between primary ovarian NETs and metastatic disease, are critical for prognosis and treatment planning.

Given the lack of prospective trials and the predominance of single-institution retrospective studies, the development of evidence-based management algorithms remains limited. Future efforts should prioritize multicenter registries and collaborative studies to standardize classification, diagnostic criteria, and treatment pathways. Until then, multidisciplinary assessment and individualized therapeutic strategies remain essential to optimize outcomes in this rare and complex clinical entity.

## 5. Conclusions

Ovarian neuroendocrine neoplasms are rare and heterogeneous tumors that present significant diagnostic and therapeutic challenges. Because of their low incidence, clinical management is largely empirical and often extrapolated from experience with extra-ovarian NETs. Accurate histopathological evaluation, including immunohistochemistry and grading, is crucial for appropriate treatment planning. There is a clear need for multicenter studies and standardized protocols to strengthen the evidence base and better guide clinical practice. Until such data are available, individualized, multidisciplinary management remains the cornerstone of care.

## Figures and Tables

**Table 1:** Main differences between 2014, 2020, and 2022 WHO classifications of neuroendocrine neoplasms.

WHO Classification	Category	Tumor
2014	Monodermal teratoma and somatic-type tumors from a dermoid cyst Miscellaneous tumors Miscellaneous tumors No category	Carcinoid Small cell ovarian NEC pulmonary type paraganglioma Small cell ovarian NEC hypercalcemia type
2020	Neuroendocrine neoplasms	Carcinoid: Grade 1 Carcinoma: Grade 3
2022	Neuroendocrine neoplasms	NET, well-differentiated: Grade 1 Grade 2 Grade 3 NEC, poorly differentiated: Small cell NEC Large cell NEC

## Data Availability

Not applicable.

## Abbreviations

The following abbreviations are used in this manuscript:

NEN	Neuroendocrine neoplasms
NET	Neuroendocrine tumors
NEC	Neuroendocrine carcinoma
SCCOPT	Small cell carcinoma of the ovary pulmonary type
SCCOHT	Small cell carcinoma of the ovary hypercalcemia type
LCNEC	Large cell neuroendocrine carcinoma of the ovary
